# Tracing the Dynamical Genetic Diversity Changes of Russian Livni Pigs during the Last 50 Years with the Museum, Old, and Modern Samples

**DOI:** 10.3390/ani14111629

**Published:** 2024-05-30

**Authors:** Alexandra A. Abdelmanova, Tatiana E. Deniskova, Veronika R. Kharzinova, Roman Yu Chinarov, Oksana I. Boronetskaya, Johann Sölkner, Gottfried Brem, Huashui Ai, Lusheng Huang, Vladimir I. Trukhachev, Natalia A. Zinovieva

**Affiliations:** 1 L.K. Ernst Federal Research Center for Animal Husbandry, Dubrovitsy, Podolsk Municipal District, Moscow Region, Podolsk 142132, Russia; preevetic@mail.ru (A.A.A.); veronika0784@mail.ru (V.R.K.); roman_chinarov@mail.ru (R.Y.C.); 2 Museum of Livestock, Timiryazev Russian State Agrarian University—Moscow Agrarian Academy, 49, ul. Timiryazevskaya, Moscow 127550, Russia; oboronetskaya@mail.ru (O.I.B.); rector@rgau-msha.ru (V.I.T.); 3 Division of Livestock Sciences, University of Natural Resources and Life Sciences, 1180 Vienna, Austria; johann.soelkner@boku.ac.at; 4 Institute of Animal Breeding and Genetics, University of Veterinary Medicine (VMU), Veterinärplatz, 1210 Vienna, Austria; gottfried.brem@vetmeduni.ac.at; 5 National Key Laboratory for Swine Genetic Improvement and Germplasm Innovation, Ministry of Science and Technology of China, Jiangxi Agricultural University, Nanchang 330045, China; aihsh@hotmail.com (H.A.); lushenghuang@hotmail.com (L.H.)

**Keywords:** pig, Livni breed, ancient DNA, genetic diversity, population structure, single nucleotide polymorphism

## Abstract

**Simple Summary:**

Simple Summary: Local breeds of pigs often do not require special housing and feeding conditions. This makes it possible to obtain high-quality meat products under eco-friendly conditions. The Livni breed is a local Russian pig breed, which is the only one adapted to pasture-based housing and produces pork of excellent quality in environmentally friendly conditions. We evaluated the genetic diversity of the Livni breed in comparison with the Large White and Landrace breeds and investigated changes in the Livni breed populations’ structure over the period from the 1970s to the present, using whole-genome single nucleotide polymorphisms. Both the museum and modern Livni pig populations are characterized by higher genetic diversity and were subject to less selection pressure than commercial pig populations. Present-day populations of the Livni pigs have retained their historical genomic components despite the use of commercial breeds in breeding efforts. This suggests their potential suitability for future breeding programs.

**Abstract:**

The pig industry is usually considered an intensive livestock industry, mainly supported by hybrid breeding between commercial pig breeds. However, people’s pursuit of a more natural environment and higher meat quality has led to an increasing demand for eco-friendly and diverse pig feeding systems. Therefore, the importance of rearing and conserving local pig breeds is increasing. The Livni pig is a local breed with good adaptability to the environmental and fodder conditions in central Russia. In this study, we aimed to analyze the genetic diversity and population structure of Livni pigs using whole-genome single nucleotide polymorphism (SNP) data. We utilized the Porcine GGP HD BeadChip on genotype samples from old (*n* = 32, 2004) and modern (*n* = 32, 2019) populations of Livni pigs. For the museum samples of Livni pigs (*n* = 3), we extracted DNA from their teeth, performed genomic sequencing, and obtained SNP genotypes from the whole-genome sequences. SNP genotypes of Landrace (*n* = 32) and Large White (*n* = 32) pigs were included for comparative analysis. We observed that the allelic richness of Livni pigs was higher than those of Landrace and Large White pigs (*A_R_* = 1.775–1.798 vs. 1.703 and 1.668, respectively). The effective population size estimates (*N_E_*_5_ = 108 for Livni pigs, *N_E_*_5_ = 59 for Landrace and Large White pigs) confirmed their genetic diversity tendency. This was further supported by the length and number of runs of homozygosity, as well as the genomic inbreeding coefficient (almost twofold lower in Livni pigs compared to Landrace and Large White pigs). These findings suggest that the Livni pig population exhibits higher genetic diversity and experiences lower selection pressure compared to commercial pig populations. Furthermore, both principal component and network tree analyses demonstrated a clear differentiation between Livni pigs and transboundary commercial pigs. The TreeMix results indicated gene flow from Landrace ancestors to Livni pigs (2019) and from Large White ancestors to Livni pigs (2004), which was consistent with their respective historical breeding backgrounds. The comparative analysis of museum, old, and modern Livni pigs indicated that the modern Livni pig populations have preserved their historical genomic components, suggesting their potential suitability for future design selection programs.

## 1. Introduction

Since the middle of the 20th century, the pig industry has focused on the development of a few highly specialized and productive pig breeds, which have been crossed to produce market-ready pigs [1,2]. The approach of terminal crossbreeding is widely accepted and recommended in commercial swine production worldwide, including Russia. As a consequence, the utilization of native pig breeds for pork production has significantly declined [3]. In Russia, nineteen locally derived pig breeds and populations have been officially recognized [4]. The majority of these local pig breeds have been developed by crossbreeding with high-performing Western pig breeds to improve their productivity [4,5]. Usually, local pig breeds exhibit commendable adaptability to local environmental conditions, extended productive lifespans, and increased growth and meat quality. From a genetic perspective, local pig breeds retain unique forms of genetic variability that have been lost in commercial pig breeds due to long-term selection exclusively focused on a limited number of traits [6,7,8,9,10].

The Livni pig, named after a small town in the Oryol region of Central Russia, is a local pig breed used for pork production during the time of the USSR. This breed was developed based on a local crossbred population that existed before 1917. It was formed through the random crossing of indigenous slow-growing pigs with Berkshire, Large White, Middle White, Lincoln, Tamworth, and Poland China boars [11,12]. Livni pigs exhibit good adaptability to the natural and feed conditions in central Russia. They are capable of thriving on pasture and have the ability to consume large amounts of cost-effective wet fodder such as potatoes, beets, green grass, and waste from grain production. Livni pigs are in high demand among citizens because of their excellent meat quality [13]. In the 1930s, there was an introduction of Large White pigs into the Livni pig breeding zone to enhance the local swine population; however, this measure was objected to by peasants as the resulting crossbreeds spoiled the turf and were unable to utilize pastures effectively [14]. At the beginning of World War II, a total of 37 boars and 200 sows were evacuated to the east of the country to preserve the breed. By 1943, 655 pigs were brought back to the Orel region, and Livni pigs were officially recognized as a breed in 1949 [14]. During the next 50 years, genetic advancement in this breed was achieved through line breeding without incorporating other breeds. In the 2000s, a portion of the purebred population was crossbred with Landrace boars to increase meat productivity and improve carcass traits [15].

During the period from 1960 to 2003, there was a substantial decrease in the population of Livni pigs, with numbers plummeting from 476,900 to 69,300 individuals [3]. Over the last two decades, the population size of Livni pigs has continued to decrease due to their lower productivity and inability to compete with commercial pig breeds. Currently, there are only 548 Livni pigs remaining in the gene pool population, comprising 29 boars, 348 sows, 6 young boars, and 165 replacement gilts, being bred on a single farm in the Oryol region [16]. Therefore, it is crucial to develop sustainable breeding programs that ensure the preservation of this valuable national genetic resource. The first step in such initiatives is the identification and preservation of specific genomic components of the original Livni pig breed.

Recent advances in high-throughput genotyping technologies allow the simultaneous analysis of tens and even hundreds of thousands of single nucleotide polymorphisms (SNPs). These genotyping technologies provide valuable insights into whole-genome variations and enable researchers to estimate the genetic diversity, population structure, and genetic relationships among different breeds of diverse animal species, including pigs [8,17,18,19,20,21]. Additionally, by examining specimens collected and stored in museums, the informative value of studies aimed at reconstructing the origin of a breed and tracking changes in genetic diversity parameters can be significantly increased [22,23,24,25,26]. Furthermore, recent success in the development of protocols for effective DNA extraction from ancient and historical samples [27] has enabled the molecular genetic analysis of such samples using different tools [28,29].

In the present study, we utilized Porcine GGP HD BeadChip to generate genome-wide SNP genotypes for a total of 143 pigs, including 67 Livni pig samples, 64 Western commercial pigs, and 12 wild boars. The Livni pig samples included three museum samples dating back to 1970–1971, 32 old samples collected in 2004, and 32 modern samples collected in 2019. We aimed to analyze the alterations in genetic diversity parameters and population structure and to trace the maintenance of original genomic components in the modern Livni pig population. The data obtained here can be valuable in selecting individuals for gene banks and enhancing the effectiveness of conservation programs for animal genetic resources.

## 2. Materials and Methods

### 2.1. Ethics Statement

The current study does not involve any endangered or protected animals, and all procedures were conducted according to the ethical guidelines of the L.K. Ernst Federal Science Center for Animal Husbandry. Protocol No. 5 from 17 July 2023 was approved by the Commission on the Ethics of Animal Experiments of the L.K. Ernst Federal Science Center for Animal Husbandry.

The tissue samples of the Livni pig samples collected in 2004 and in 2019 as well as the samples of Western commercial pigs were collected by trained personnel under strict veterinary rules in accordance with the rules for conducting laboratory research (tests) in the implementation of the veterinary control (supervision) approved by Eurasian Economic Commission Council Decision No. 80 (10 November 2017) during the work on the herd. The muscle tissue samples of the wild boar came from regular hunting, according to the Russian Federation Law No. 209-FZ of 24 July 2009.

### 2.2. Sample Collection and DNA Extraction

**Figure 1:**
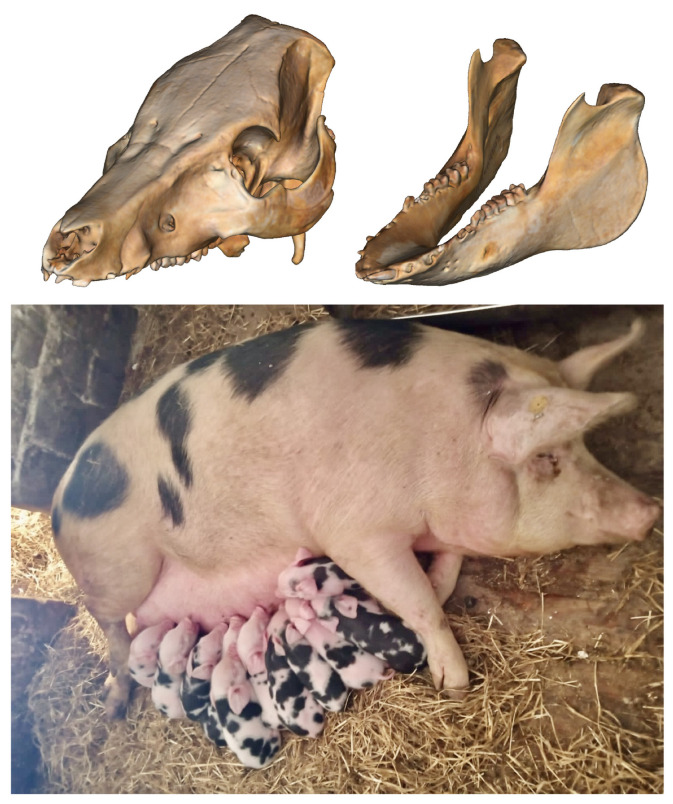
Livni pigs. The skull of the Livni pig, museum sample, 1970 (top figure) (photo provided by Nikolaev A.); Livni sow with piglets, present time (bottom figure) (photo provided by Shelamova N.).

All samples used in this study were obtained from Livni pigs (Figure 1) bred on a single breeding farm in the Orel region.

Samples of Livni pigs dated to the years 1970–1971 of the 20th century (H_LV, *n* = 3) were derived from swine skulls stored in the Museum of Livestock (Moscow Agricultural University named after K.A. Timiryazev) (Figure 1).

The samples of Livni pigs dated to 2004 (LV_2004, *n* = 32) and 2019 (LV_2019, *n* = 32), as well as the Western commercial pigs (samples of Landrace (LN, *n* = 32) and Large White (LW, *n* = 32) breeds) and wild boars (WB, *n* = 12), were obtained from the Collection of Animal Genetic Resources of the L.K. Ernst Federal Research Center for Animal Husbandry.

All experiments involving museum samples were performed in the facility of the L.K. Ernst Federal Research Center for Animal Husbandry, which specializes in ancient DNA studies. The teeth were removed from the skull, and the tooth roots were sawed off using an MBS 240/E electric saw (Proxxon, Trier, Germany). Bone powder was then produced using a Retsch MIXER MILL MM 400 (Retsch GmbH, Haan, Germany). Genomic DNA was extracted from a total of 200 ± 5 µg of bone powder, using a COrDIS Extract Decalcine Kit (GORDIZ LLC, Moscow, Russia) in accordance with the manufacturer’s instructions, with several modifications of the lysis conditions and volumes (lysis at 56 °C, 1200 rpm, overnight, using a 2.5× volume of lysis and washing buffers). Additionally, a negative control tube (reagent blank) was prepared, containing only DNA extraction reagents, to trace any potential DNA contamination.

Genomic DNA of the Livni pigs dated to 2004 and 2019, the Western commercial pigs, and wild boars was extracted from the tissue of each animal using NEXTTEC kits (Nexttec Biotechnologie GmbH, Hilgertshausen, Germany) and “DNA-extran-2” (OOO ‘Syntol’, Moscow, Russia), according to the manufacturers’ protocols.

To evaluate the quantitative and qualitative characteristics of the DNA solutions, the concentration of double-stranded DNA was measured using a Qubit™ fluorometer (Invitrogen, Life Technologies, Carlsbad, CA, USA), and the absorption at 260 and 280 nm (OD 260/280) was determined using a NanoDrop 8000 instrument (Thermo Fisher Scientific Inc., Waltham, MA, USA). Before further processing, the DNA extracted from the museum samples was treated with 1 unit of USER enzyme (NEB, Hitchin, UK) and then incubated overnight at 37 °C. This step was aimed at removing uracil residues resulting from post-mortem DNA damage [30], in accordance with the manufacturer’s instructions.

### 2.3. SNP Genotyping and Quality Control

Genotyping for the samples from the modern pig population was performed using the GeneSeek^®^ Porcine GGP HD BeadChip (Illumina, San Diego, CA, USA). Input files to further analyze by PLINK 1.9 software [31] were created using R 3.5.3 software [32].

Genotyping of museum samples by the GeneSeek^®^ Porcine GGP HD BeadChip (Illumina, San Diego, CA, USA) was low efficient, with an average of 69.79% of SNPs successfully genotyped. To improve the genotyping, whole genomes of the museum samples were sequenced, and these sequences were used to create a virtual SNP chip with positions identical to those presented on the GeneSeek^®^ Porcine GGP HD BeadChip. Then, relevant data from the GeneSeek^®^ Porcine GGP HD BeadChip and from the created virtual SNP chip for each museum sample were merged to maximize the number of successfully genotyped SNPs. As a result, the average proportion of SNPs successfully genotyped increased to 84.28%.

To determine the valid genotypes for each SNP, we set a cut-off score of 0.5 for GenCall and GenTrain [33]. SNP quality control was performed using PLINK 1.9 software [31]. We only included animals that passed the filtering criteria of more than 75% genotyping efficiency (--mind 0.25). Only SNPs located on autosomes and genotyped in more than 90% of the samples (--geno 0.1) were used for further analysis. This resulted in a final dataset consisting of 52,706 autosomal SNPs. For linkage disequilibrium (LD) filtering, one SNP from each pair of neighboring SNPs, where the LD (r2) value exceeded 0.5 within 50 SNP windows, was removed using --indep-pairwise 50 5 0.5 flag, where 50 is the size of the sliding window, 5 is the number of SNPs shifted in each step, and 0.5 is the r2 threshold. In total, 25,987 SNPs successfully passed through the LD filter and were used for genetic diversity calculation, principal component analysis (PCA), neighbor-net tree construction, admixture clustering, and TreeMix analysis.

SNP genotypes of the wild boars involved in this study were taken from the bioresource collection of the L.K. Ernst Federal Research Center for Animal Husbandry.

### 2.4. Genetic Diversity and Effective Population Size

Within-population genetic diversity was evaluated using PLINK 1.9 [31]. We calculated the observed heterozygosity (*H_O_*), unbiased expected heterozygosity (*_U_H*_E_) [34], rarefied allelic richness (*A_R_*) [35], and inbreeding coefficient (*_U_F*_IS_) based on unbiased expected heterozygosity using the R package diveRsity [36].

To estimate trends in the effective population size, the LD was analyzed using the SNeP tool, as previously described by Barbato et al. [37]. Default parameters were utilized, except for sample size correction, mutation occurrence (α = 2.2) [38], and recombination rate, between a pair of genetic markers, as described by Sved and Feldman [39]. To determine the rate of *N_E_* change in the 50 most recent generations, the *N_E_* changing ratios were determined by calculating the slope of each segment that links a pair of neighboring N_E_ estimates. The values were then normalized using the median of the most recent 50 N_E_ estimates. For the estimation of effective population size, only SNPs with a minor allele frequency (MAF) greater than 0.05 were used.

We did not estimate the N_E_ values for the H_LV and LV_2004 groups, as they cannot be statistically compared to those calculated for the LN and LW groups genotyped in 2020.

### 2.5. Runs of Homozygosity (ROHs)

We used a window-free method for the consecutive SNP-based detection of ROHs [40] implemented in the R package detectRUNS [41]. One SNP with missing genotype and up to one possible heterozygous genotype in one run was allowed to avoid underestimating the number of ROHs that were longer than 8 Mb [42]. We set the minimum length for the ROHs to 1000 kb to exclude short and common ROHs. For minimizing false-positive results, the minimum number of SNPs (*l*) was calculated, as was initially conducted by Lencz et al. [43] and later modified by Purfield et al. [44]:

$$l=\frac{{log}_{e\frac{\alpha }{n_{s}*n_{i}}}}{{log}_{e}(1-\bar{het})}$$

where *n_s_* is the number of genotyped SNPs per individual, *n_i_* is the number of genotyped individuals, *α* is the percentage of false-positive ROHs (set to 0.05 in our study), and $\bar{het}$ is the mean heterozygosity across all SNPs. In our case, the minimum number of SNPs was 20.

Based on the genome coverage by ROHs, we computed the genomic inbreeding coefficient (FROH) as the ratio of the sum of the lengths of all ROHs per animal to the total autosomal SNP coverage (2.45 Gb). Additionally, we examined the number of ROHs in the genomes of the studied breeds by length classes (1–2, 2–4, 4–8, 8–16, and >16 Mb) and the proportion of the genome covered by ROH segments of different minimum lengths (>0.5, >1, >2, >4, >8, and >16 Mb).

### 2.6. PCA, Neighbour-Net, and Admixture

PCA was performed using PLINK v1.9 software [31], and the results were visualized using the R package ggplot2 [45]. Model-based clustering was performed using Admixture v1.3 software [46], and the R package BITE [47] was used to plot the results. We used a standard admixture cross-validation procedure [48] to calculate the number of ancestral populations (K). We tested K values from 1 up to 5, and the most probable K values corresponding to the lowest value of cross-validation error, as compared with the other K values, was two (Figure S1A).

### 2.7. TreeMix Analysis

TreeMix analysis was performed using TreeMix 1.12 [49] to investigate population splits and the extent of gene exchange among the studied breeds. Wild boars were defined as an outgroup population to set the root position in the maximum likelihood tree produced using TreeMix. Up to five migration events in 10 iterations per migration edge were tested, and the optimal number of migration events was determined based on TreeMix output files using the R package ‘optM’ (Figure S1B) [50]. Furthermore, the F-index was calculated to assess the contribution of each migration vector to the tree.

### 2.8. D-Statistics and F-Statistics

D-statistics [51], F3-, and F4-statistics [52] were computed to investigate the genetic relationships and historical admixture patterns between the studied breeds using the R package ‘admixr’ [53]. For a case of phylogeny (((W, X), Y), O), we used wild boars as outgroup O. In two alternative analyses, we defined LW or LN pigs as Y because of their possible contribution to the development of Livni pigs. We tested all possible combinations of the studied populations of Livni pigs (H_LV, LV_2004, LV_2019) as W and X. Additionally, to evaluate the contribution of the museum population to the genetic pool of old and modern Livni pigs, we tested the museum population as Y, the old or modern populations as W, and Landrace as their sister breed X. To trace the gene flow between the old and modern populations of Livni pigs, we defined the old population of Livni pigs as Y, the modern population as W, and Landrace as X. D-values and F4-values with Z scores > 3 were considered statistically significant.

To estimate the proportion of possible ancestry in the Livni pigs, we performed an F4-ratio test [52]. We defined each of the studied livestock populations of Livni pigs as an introgressed population X. Source population B was defined based on the results of D-statistics. Wild boars were used as outgroup O. An ancestry proportion of B in X (α) with Z scores > 3 was considered statistically significant.

To understand the relationships among the studied breeds, we used F3-statistics (A, B, and C) [52]. For admixture F3-statistics, we defined each of the studied populations of Livni pigs as the target population (C) and tested the LN, LW, and earlier populations of Livni pigs as source populations (A and B) for the following pairs of breeds: the museum population was used as the source population for LV_2004 and LV_2019; LV_2004 was used as the source population for LV_2019. The negative F3-values with Z scores ≤ −3 suggests a significant rejection of the null hypothesis that the statistic is not negative and indicates admixture between the two source populations. To test the closest of the studied populations of Livni pigs to each other and to commercial pig breeds, we implemented outgroup F3-statistics, where we defined wild boars as outgroup C and the studied breeds as tested populations A and B. Higher F3-values indicate more genetic similarities between A and B.

## 3. Results

### 3.1. Genetic Diversity and Effective Population Size

The allelic richness in all groups of the Livni pigs was higher than that in the LN and LW pigs (1.775–1.798 vs. 1.703 and 1.668, respectively) (Table 1).

Significant heterozygote excess was observed in the LN and LW pigs (*_U_F*_IS_ = −0.038 and *_U_F*_IS_ = −0.033, respectively). Heterozygous excess was also found in the modern Livni pigs, but it was almost three times lower than that in the commercial pigs. Heterozygotes were lacking in the museum samples of the Livni pigs, which may be attributed to the limited sample size.

**Figure 2:**
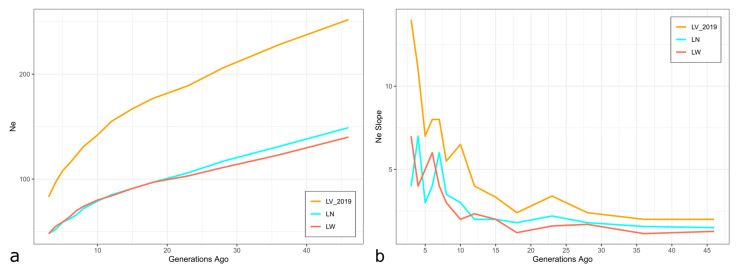
The effective population size (*N_E_*) across generations from approximately 50 generations ago based on linkage disequilibrium (LD) calculations (**a**) and *N_E_* slope (**b**) for studied swine populations: LV_2019—Livni breed, dated by 2019 years, LN—Landrace, LW—Large White.

The *N_E_* values of the studied breeds decreased over time. Five generations ago the Livni pigs had a larger effective population size (*N_E_*_5_ = 108) than the commercial pigs (*N_E_*_5_ = 59 for both LN and LW) (Figure 2a,b).

### 3.2. Patterns of ROH Distribution

LV_2004 and LV_2019 had similar patterns of ROH distribution (Table 2), with mean ROH lengths of 431.20 and 451.57 Mb, respectively, and ROH numbers of 216.06 and 213.62, respectively.

Comparison with transboundary breeds revealed that the Livni pigs had lower ROH numbers than the commercial pigs (213.62–216.06 vs. 249.53 in LN and 285.59 in LW) and smaller mean ROH lengths (313.51–451.57 Mb vs. 686.13 in LN and 792.78 in LW). Furthermore, the maximal ROH lengths in the Livni pigs were lower than those in the LN and LW pigs. H_LV had a smaller ROH length and FROH than LV_2004, LV_2019, LN, and LW. It also had a higher ROH number than LV_2004 and LV_2019, indicating an increase in inbreeding in this breed. The FROH values of the Livni pigs were twice as low as those of the LN and LW pigs (FROH = 0.128–0.184 vs. 0.280 and 0.324 in LN and LW, respectively).

**Figure 3:**
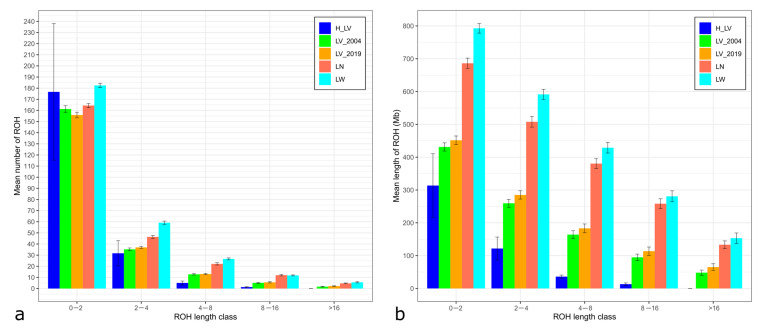
Descriptive statistics of the runs of homozygosity (ROHs) by ROH length class in the studied populations: H_LV, LV_2004, and LV_2019—Livni breed, dated by the years 1970–1971, 2004, and 2019, respectively, LN—Landrace, LW—Large White; (**a**) mean number of ROHs (*Y*-axis) by ROH length class (*X*-axis; 0.5–2, 2–4, 4–8, 8–16, and >16 Mb); (**b**) overall mean length of ROHs (*Y*-axis) by ROH length class (*X*-axis; >0.5 Mb, >2 Mb, >4 Mb, >8 Mb, and >16 Mb).

Short ROH segments were predominant in all Livni populations as well as in the LN and LW breeds (Figure 3).

In addition, the highest coverage in ROHs was attributed to the length class.

### 3.3. Genetic Differentiation and Admixture Patterns in the Studied Pig Populations

**Figure 4:**
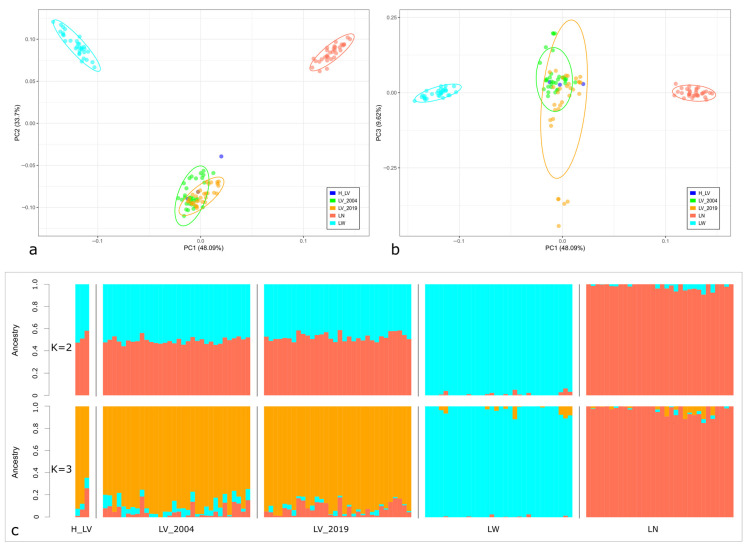
Genetic relationships between the studied populations: H_LV, LV_2004, and LV_2019 —Livni breed, dated by the years 1970–1971, 2004, and 2019, respectively, LN—Landrace, LW—Large White. (**a**) Principal Component Analysis (PCA) plot showing the distribution of individuals of studied breeds in the dimensions of two coordinates; *X*-axis—principal component 1 (PC1); *Y*-axis—principal component 2 (PC2); (**b**) *X*-axis—principal component 1 (PC1); *Y*-axis—principal component 3 (PC3), the percentage of total genetic variability, which can be explained by each of the two components, being indicated within the parentheses; (**c**) an Admixture plot representing the cluster structure of the studied populations for the number K from 2 to 3.

A study of the genetic relationships between pig populations showed that PC2 accounted for 33.7% of genetic variability separating the Livni breed from both transboundary breeds (Figure 4a).

In the PC1–PC2 dimension, Livni pigs dated to different periods formed a joint cluster, except for one museum sample demonstrating low differentiation. However, PC3 accounted for 9.62% of total genetic variability, demonstrating that several specimens collected in 2004 and 2019 formed separate groups (Figure 4b). A detailed examination of these samples showed a high proportion of genomic components, which were characteristic of transboundary breeds, averaging 22.35% (from 18.1% to 26.2%), whereas the share of Livni components was, on average, less than 80% (Figure 4c, Table S1). At the same time, LV_2004 and LV_2019 preserved components that were predominant in the museum specimens. Notably, a higher proportion of LN components was observed in the samples from LV_2019 than in those from LV_2004 (7.54% vs. 5.10%). However, the genetic contributions of the transboundary breeds to the genomic composition of the samples from LV_2004 were approximately equal (5.10% for LN and 5.94% for LW).

### 3.4. TreeMix Analysis

**Figure 5:**
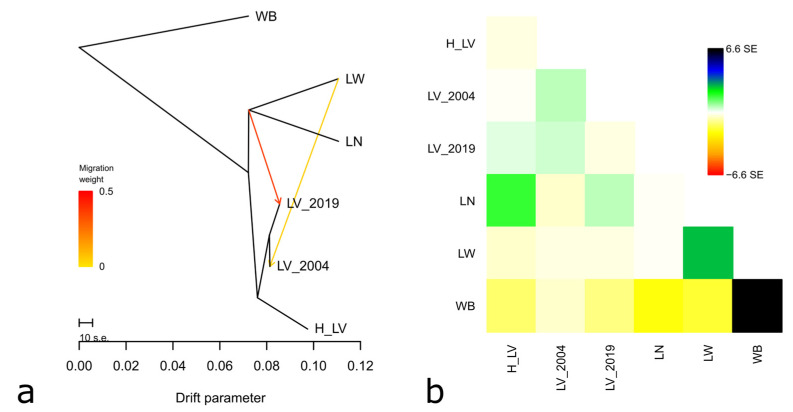
TreeMix tree with two migration events and the Asian wild boar fixed as the root; the scale bar shows 10 times the average standard error (s.e.) of the estimated entries in the sample covariance matrix. Putative gene flow is indicated by the arrows, pointing in the direction of flow from the donor to the recipient population, and colored in orange-red, proportional to the gene flow intensity (**a**); residual matrix plotted from a TreeMix analysis under two migration events and expressed as the number of standard errors (s.e.) of the deviation (**b**); studied pig populations: H_LV, LV_2004, and LV_2019—Livni breed, dated by the years 1970–1971, 2004, and 2019, respectively, LN—Landrace, LW—Large White, WB—wild boar.

In the TreeMix tree, all studied populations of Livni pigs were localized on a joined branch, which provided evidence of their common ancestry (Figure 5).

These two migration events were optimal (Supplementary Figure S1B). The migration vector clearly indicated gene flow from LN to LV_2019 and from LW to LV_2004, which is in accordance with the breed history.

### 3.5. D-Statistics and F-Statistics

The D-statistics results showed the contribution of the LW and LN breeds to the development of the studied populations of Livni pigs (Z-score > 3). Testing the museum population as Y pointed to gene flow in LV_2004 and LV_2019, which confirmed the common origin of all these populations (Table S2A).

The F4-statistics results showed that the number of shared alleles with the museum population was higher in LV_2004 than in LV_2019 (Table S2B).

Compared with LW, LV_2019 had more common SNPs shared with the museum specimens. However, compared with LN, LV_2004 had more common SNPs shared with the museum specimens (Table S2B). These results indicated that the breeding of Livni pigs was multidirectional. The Z-score for the D-value in the case of phylogeny (((LV_2004, LN), LV_2019) WB) was 5.599, indicating a genetic exchange between the old and modern populations of Livni pigs (Table S2A).

Admixture F3-statistics did not reveal negative Z-scores of ≤−3, which can be considered as confirmation of the non-crossbred origin of Livni pigs (Table S2C). F3-statistics with wild boar as the outgroup showed that LV_2004 was slightly closer to the museum population than to LV_2019, as indicated by higher F3-values. Interestingly, the LV_2004 pigs were closer to the LW pigs than to the LN pigs, whereas the LV_2019 pigs were closest to the LN pigs (Table S2D). This result may reflect the contribution of the LN breed to the development of the modern population of Livni pigs.

## 4. Discussion

Local pig breeds are preferred by small-scale producers due to their resilience and ability to adapt to local conditions and available feed resources. Local pig breeds are well suited for low-input production systems, making them an ideal choice for small-scale farming [54]. In addition, these breeds are essential in the development of organic and slow food production systems, contributing to sustainable and environmentally friendly agricultural practices.

The Livni breed is suitable for eco-friendly farming for several reasons. Firstly, these pigs can graze without damaging the turf, which is unusual for domestic pigs. Secondly, Livni pigs are capable of satisfactory meat production by using waste and by-products from the beet and grain industries. Preserving and characterizing the genetic diversity of the Livni breed is one of the key objectives to ensure the long-term sustainability of production systems in Central Russia. Additionally, the conservation effort contributes to the national pig breeding branch by reducing the import of transboundary breeds.

Genomic studies of different populations of Livni pigs have been previously conducted using DNA chips [5,55]. Traspov et al. [5] focused on unraveling the genetic relationships between various Russian local pig breeds, while Chernukha et al. [55] performed a search to identify specific genetic loci associated with selection pressure in Livni and Duroc breeds. However, there has been a lack of comprehensive research on Livni pig populations across different periods. In our present study, we detected several genomic patterns by comparing the genetic diversity indicators of old (2004) and modern (2019) Livni populations with those of two transboundary breeds (LN and LW pigs). Firstly, the observed heterozygosity and allelic richness were higher in the Livni populations than in the transboundary breeds. Secondly, the *N_E_* estimates were higher in the Livni breeds than in the transboundary breeds. Thirdly, the genome coverage with ROH length and number was significantly lower (almost twice) in the Livni pigs than in the transboundary breeds. These observations suggest a higher genetic consolidation resulting from greater selection pressure in the LN and LW pigs compared to the local Livni pigs.

Compared with other local pig breeds, the Livni pig is characterized by comparable or lower genome inbreeding. Thus, the endangered Turopolje pig breed shows a very high inbreeding level (*F*_ROH_ = 0.400 [8] vs. 0.176–0.184 in Livni groups). *F*_ROH_ values vary from 0.100 in the Hungarian Blond Mangalitza to 0.170 in the Romanian local Bazna [56], and from 0.160 in Banija spotted to 0.185 in Krskopolje pigs in Balkan local breeds [57]. The genetic diversity indicators of Livni populations are higher than those in Eastern European local (*H_O_* = 0.31 in Bazna and *H_O_* = 0.32 in Red Mangalitza [56]) and Balkan local pigs (*H_O_* = 0.363 in Banija spotted, *H_O_* = 0.348 in Moravka, and *H_O_* = 0.366 in Krskopolje pigs) [57]. Moreover, recent *N_E_* values estimated in Livni pigs exceed those calculated in Black Slavonian (*N_E_* = 33) and Turopolje pigs (*N_E_* = 10) [8].

Based on the TreeMix results and analysis of migration events, our findings suggest that both the LV_2004 and LV_2019 Livni populations may have been improved by the transboundary breeds. Genomic components present in the LN and LW breeds were also detected in the relevant Livni populations. This pattern probably reflects an improvement in bacon traits as the main breeding objective in the development of the Livni breed. This assumption may take place because the Livni breed is traditionally of the fat type, whereas bacon is more demanded by customers and is now more highly priced. Attempts to utilize LN for meat productivity enhancement in several livestock herds have been reported recently [58].

Developing conservation and selection programs for local livestock breeds is a crucial step that ensures the preservation of diverse genetic resources for future generations. These resources play a vital role in enabling livestock to possess enhanced environmental adaptations and specific traits that can withstand future anthropogenic and environmental changes. However, considering the lasting and sometimes excessive improvement of local breeds with transboundary breeds, breeders and geneticists often face the big issue of whether the original identity has been preserved in the breed of interest or irretrievably lost. To solve this problem, the investigation of museum samples representing earlier versions of modern local breeds becomes crucial. These museum samples, such as skulls, bones, stuffed animals, and hair, serve as valuable resources for population and conservation genomic studies due to their temporal span [59].

DNA extracted from museum specimens can enable the direct comparison of temporal changes in connectivity among populations over time, which can provide valuable insights for conservation planning [60]. For example, Parejo et al. [61] conducted a study at the Natural History Museum in Bern, Switzerland, where genomic sequencing was utilized to analyze 22 bee specimens, several of which were dated 150 years old. This analysis indicated no significant genetic bottleneck or drastic decrease in the genetic diversity of bees over that time period. In addition, genetic studies on historical and modern samples have demonstrated the maintenance of historical components in modern populations of Kholmogor and Yaroslavl cattle [25]. Similarly, Kvist et al. [62] examined the history of the native horse breed Finnhorse by using historical DNA samples from before, during, and after its foundation, spanning from the end of the 19th century to the entire 20th century.

Thus, in the present study, we provided a comprehensive genetic description of the Livni breed and tried to address whether the original Livni genomic components are still present in the modern population. To achieve this goal, we performed the first comparative genomic analysis of SNP genotypes generated from historical samples of the Livni pig breed. We found that both the old and modern Livni groups joined the historical population on PCA and the neighbor-net graph. Comparative clustering of the historical, old, and modern Livni groups demonstrated similarities in their population structures. Based on the SNP data, we hypothesized that the Livni populations of 2004 and 2019 maintained the original background of historical unimproved samples.

## 5. Conclusions

We used genome-wide SNP data generated from historical museum specimens to improve the genomic analysis of the local pig breed Livni. Our results provide evidence that although modern populations of Livni pigs have been improved by commercial breeds, they preserved their historical genomic backgrounds. Thus, these results may serve as a reference for conserving and improving this local pig breed.

## Figures and Tables

**Table 1:** Summary statistics for genetic diversity calculated for the studied populations based on SNP genotypes.

^1^ Population	^2^ n	^3^ *H_O_* (^7^ M ± ^8^ SE)	^4^ *_U_H*_E_ (M ± SE)	^5^ *A_R_* (M ± SE)	^6^ *_U_F*_IS_ [CI 95%]
H_LV	3	0.383 ± 0.002	0.392 ± 0.001	1.775 ± 0.003	0.022 [0.016; 0.028]
LV_2004	32	0.409 ± 0.001	0.404 ± 0.001	1.798 ± 0.001	−0.011 [−0.013; −0.009]
LV_2019	32	0.405 ± 0.001	0.401 ± 0.001	1.793 ± 0.001	−0.009 [−0.011; −0.007]
LN	32	0.366 ± 0.001	0.351 ± 0.001	1.703 ± 0.002	−0.038 [−0.04; −0.036]
LW	32	0.345 ± 0.001	0.333 ± 0.001	1.668 ± 0.002	−0.033 [−0.035; −0.031]

^1^ Population: H_LV, LV_2004, and LV_2019—Livni breed, dated by the years 1970–1971, 2004, and 2019, respectively, LN—Landrace, LW—Large White; ^2^ n: sample size; ^3^ *H_O_*: observed heterozygosity; ^4^ *_U_H*_E_: unbiased expected heterozygosity; ^5^ *A_R_*: allelic richness; ^6^ *uF*_IS_: inbreeding coefficient based on the difference between *_U_H*_E_ and *H_O_* with a 95% confidence interval (CI; in square brackets); ^7^ M: mean value; ^8^ SE: standard error.

**Table 2:** Summary statistics for runs of homozygosity (ROHs) calculated for the studied populations based on SNP genotypes.

^1^ Population	^2^ n	^3^ ROH Length, Mb			^4^ ROH Number			^5^ F_ROH_
		^6^ M ± ^7^ SE	^8^ MIN	^9^ MAX	M ± SE	MIN	MAX	M ± SE
H_LV	3	313.51 ± 96.88	119.77	412.15	214.67 ± 71.55	73	303	0.128 ± 0.04
LV_2004	32	431.20 ± 12.58	320.43	589.78	216.06 ± 3.51	189	257	0.176 ± 0.005
LV_2019	32	451.57 ± 13.16	330.77	659.71	213.62 ± 2.81	188	240	0.184 ± 0.005
LN	32	686.13 ± 15.73	517.98	851.82	249.53 ± 2.66	220	283	0.280 ± 0.006
LW	32	792.78 ± 14.73	670.87	1059.59	285.59 ± 2.17	266	314	0.324 ± 0.006

^1^ Population: H_LV, LV_2004, and LV_2019—Livni breed, dated by the years 1970–1971, 2004, and 2019, respectively, LN—Landrace, LW—Large White; ^2^ n: sample size; ^3^ ROH length: the overall length of ROHs in a genome; ^4^ ROH number: the number of ROHs in a genome; ^5^ F_ROH_: inbreeding coefficient calculated based on genome coverage by ROHs; ^6^ M: mean value; ^7^ SE: standard error; ^8^ MIN: minimal value; ^9^ MAX: maximal value.

## Data Availability

The SNP genotypes for all the pigs and wild boars individuals used in our study are available on request from the corresponding authors.

## Supplementary Materials

The following supporting information can be downloaded at: https://www.mdpi.com/article/10.3390/ani14111629/s1; Figure S1: Estimation of the number of assumed ancestral populations and optimal number of migration events for the studied populations; Table S1: The proportion of genomic components of ancestral populations in the structure of the studied breeds; Table S2A–D: Results of D- and F-statistics.
