# Supplementary material for: Tracing the Dynamical Genetic Diversity Changes of Russian Livni Pigs during the Last 50 Years with the Museum, Old, and Modern Samples
Source: Animals (Basel). 2024 May 30;14(11):1629. doi: 10.3390/ani14111629 (PMC11171240; doi:10.3390/ani14111629)
Supplement: Supplementary file 1 [file animals-14-01629-s001.zip › Supp_Fig1AB_cverror_optM.pdf]

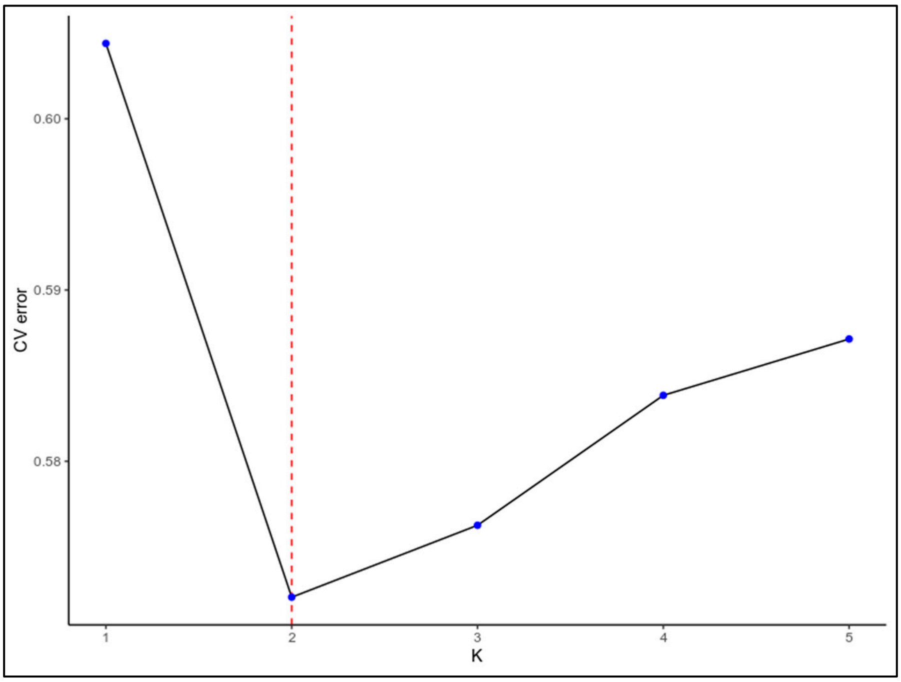

Figure S1A. Estimation of the number of assumed ancestral populations (K) on the basis of the lowest cross-validation (CV) error.

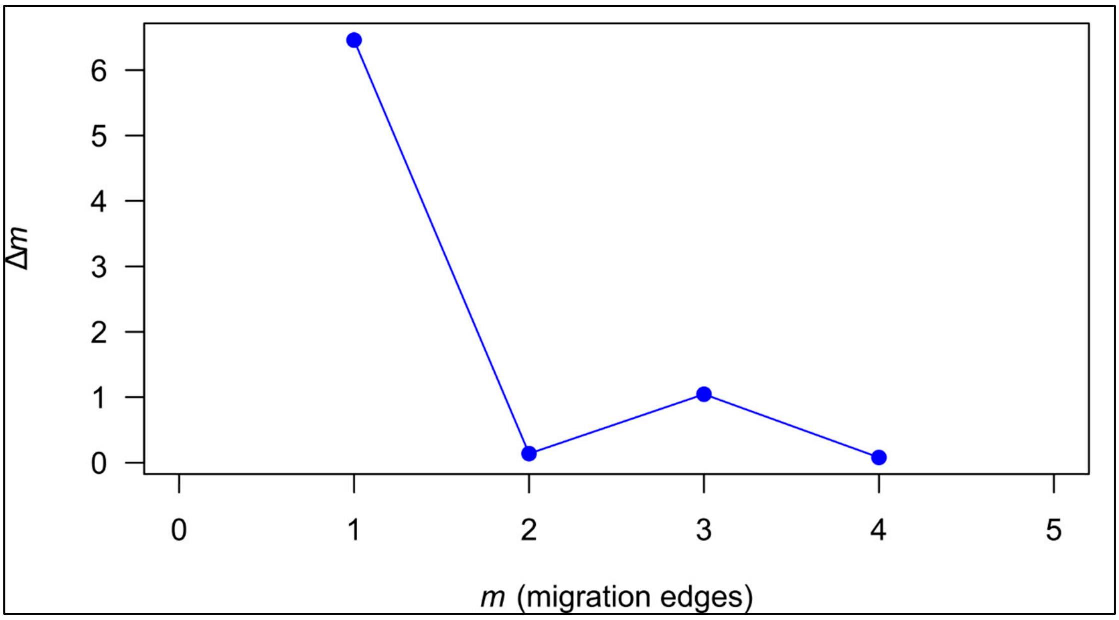

Figure S1B. Estimation of the optimal number of migration events (m) inferred from the second-order rate of change in likelihood (Δm) across incremental values of m performed using "optM" R package.
