# Supplementary figures and images for: Tracing the Dynamical Genetic Diversity Changes of Russian Livni Pigs during the Last 50 Years with the Museum, Old, and Modern Samples
Source: Animals (Basel). 2024 May 30;14(11):1629. doi: 10.3390/ani14111629 (PMC11171240; doi:10.3390/ani14111629)

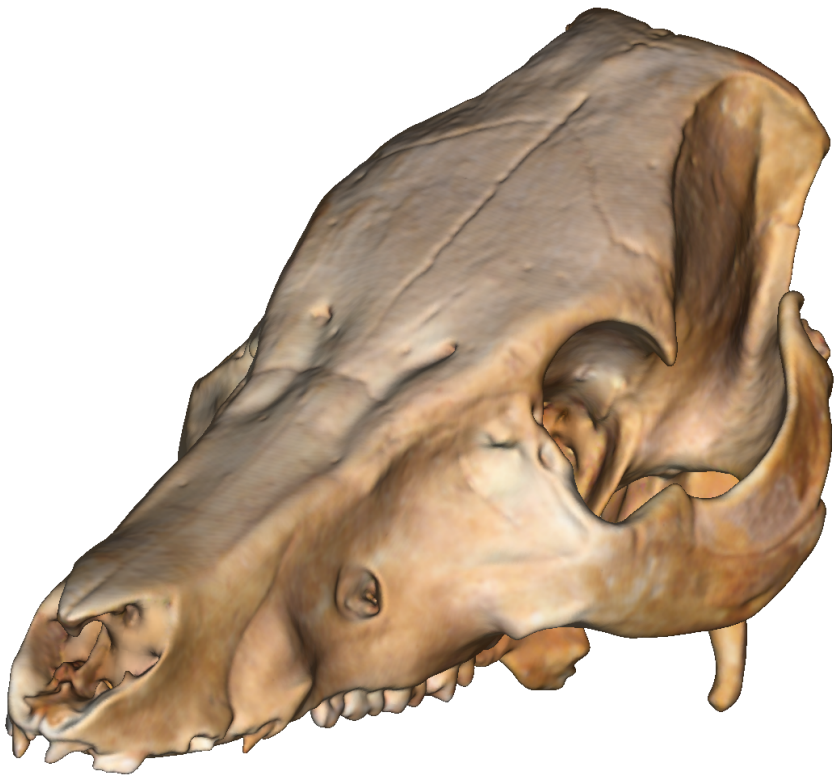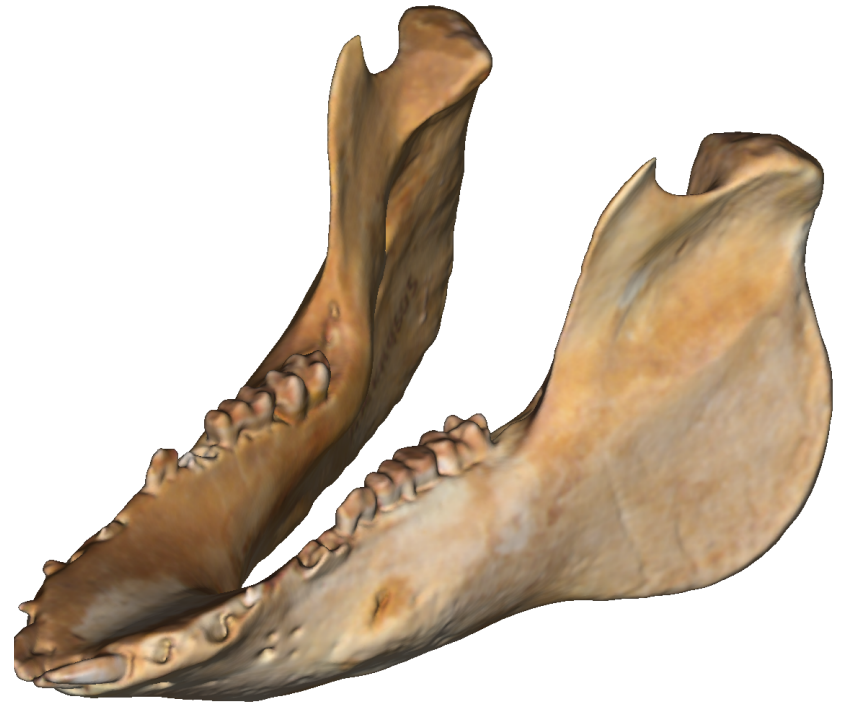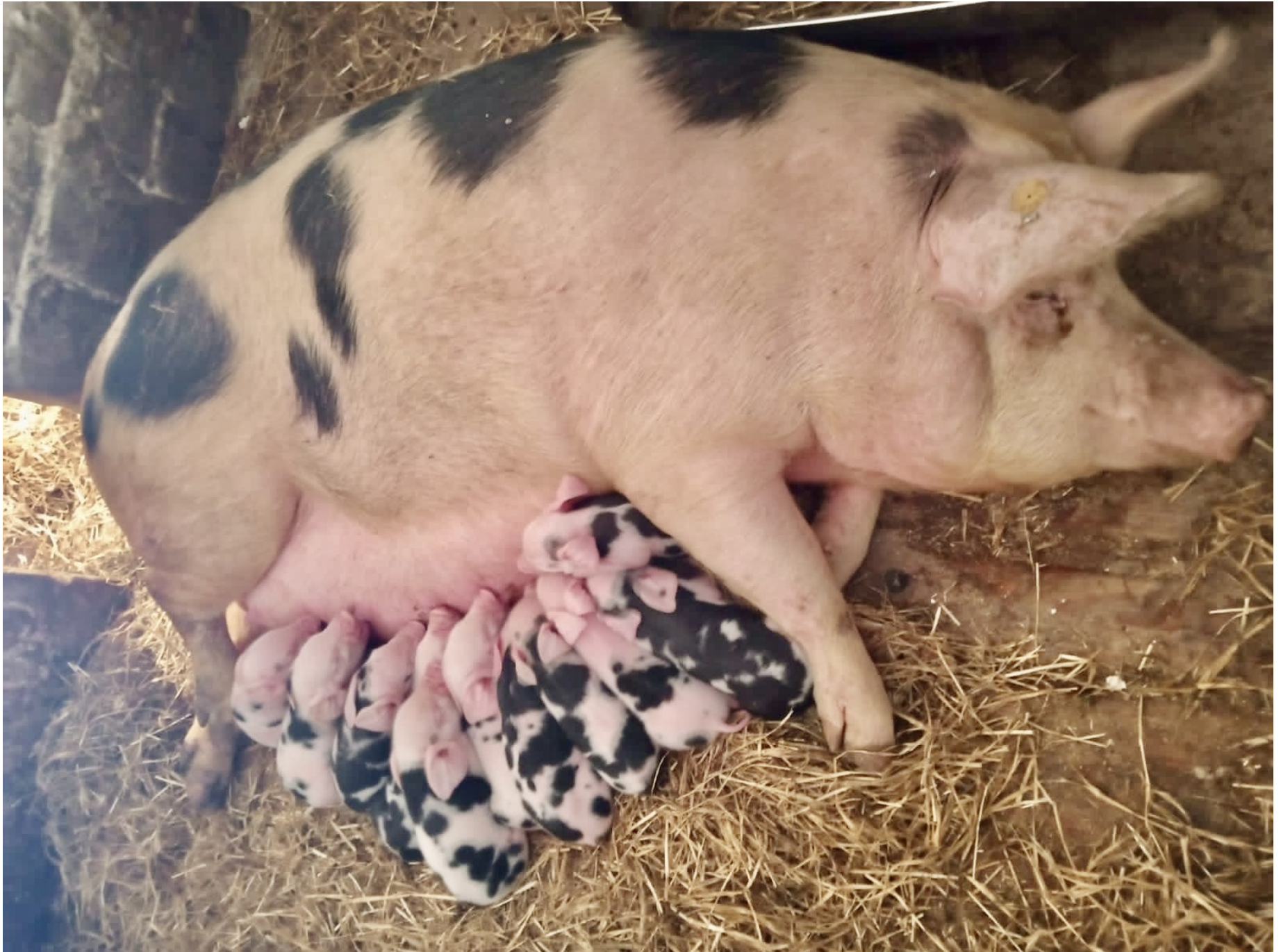

Supplement: Supplementary file 1 [file animals-14-01629-s001.zip › Fig1_Scull_and_piglets2.pdf]

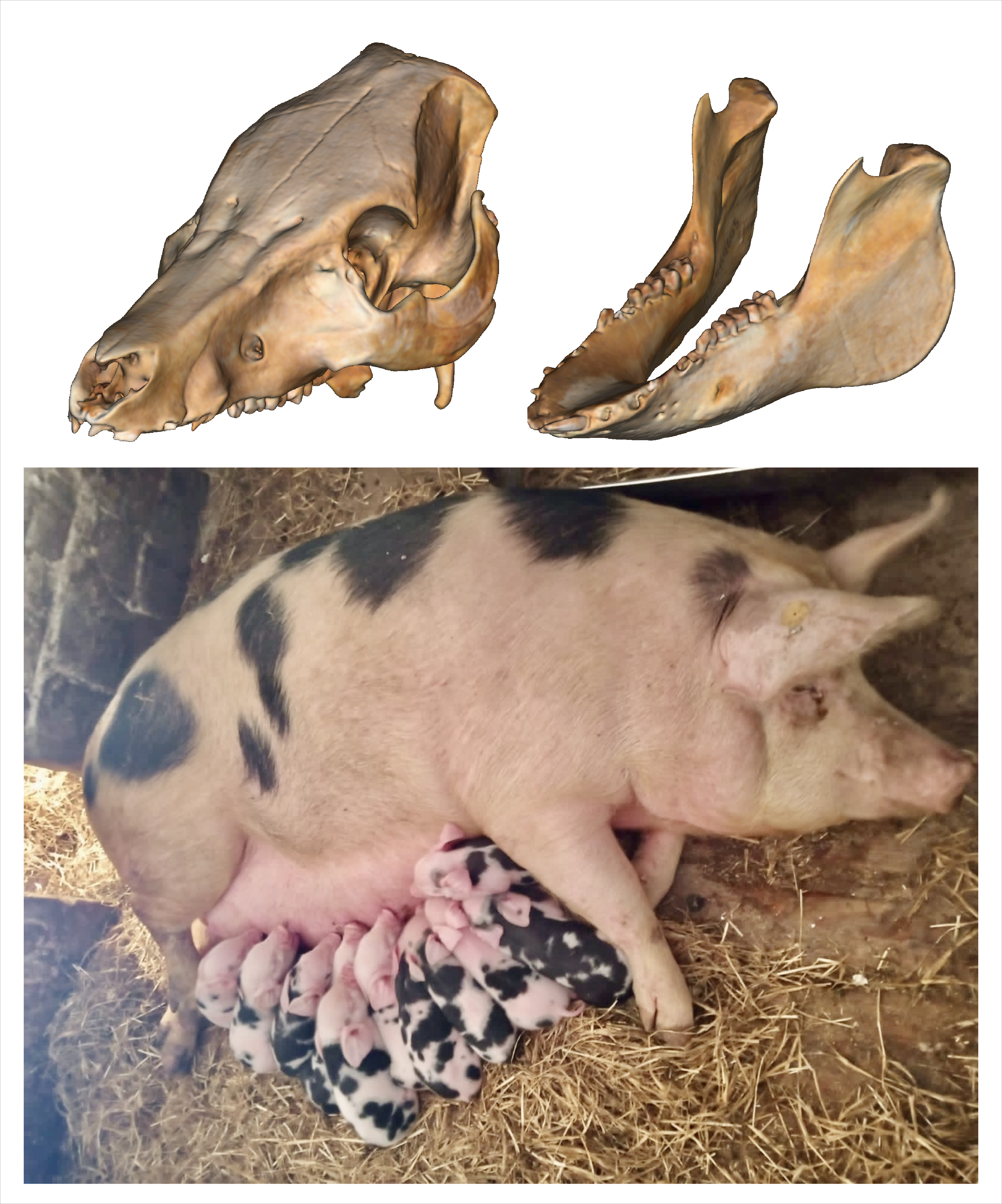

Supplement: Supplementary file 1 [file animals-14-01629-s001.zip › Fig1_Scull_and_piglets2.png]

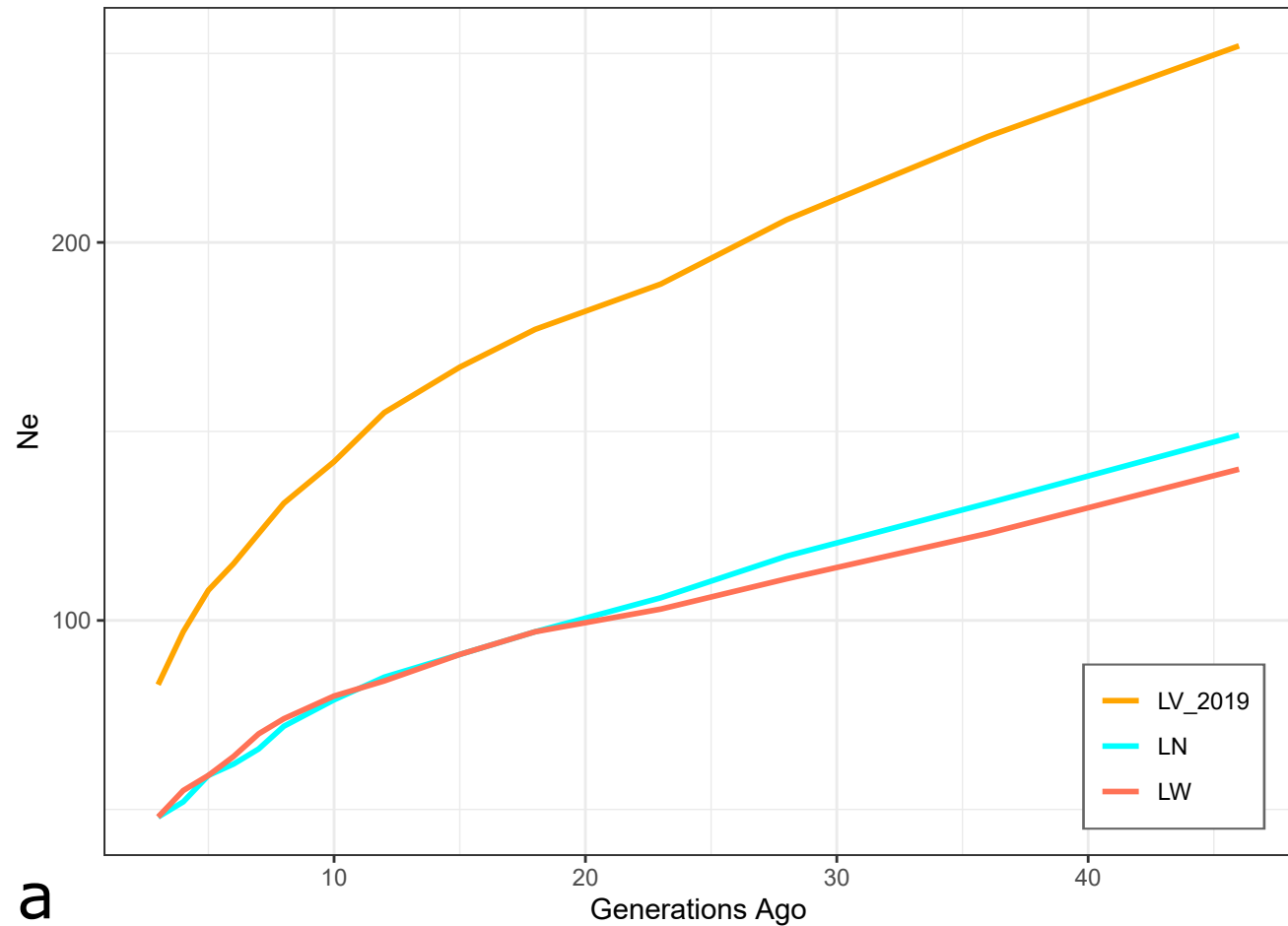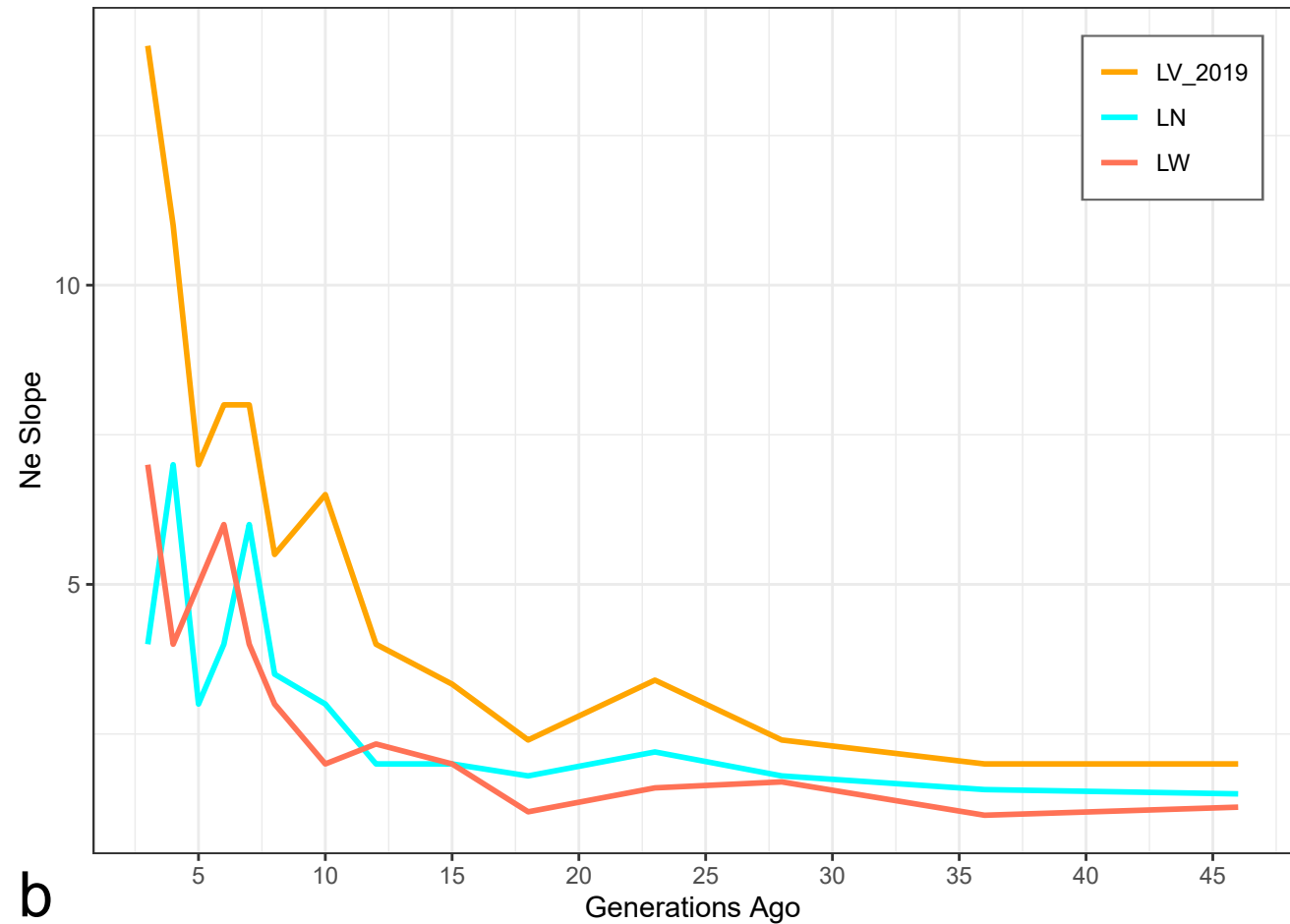

Supplement: Supplementary file 1 [file animals-14-01629-s001.zip › Fig2_Ne_Neslope.pdf]

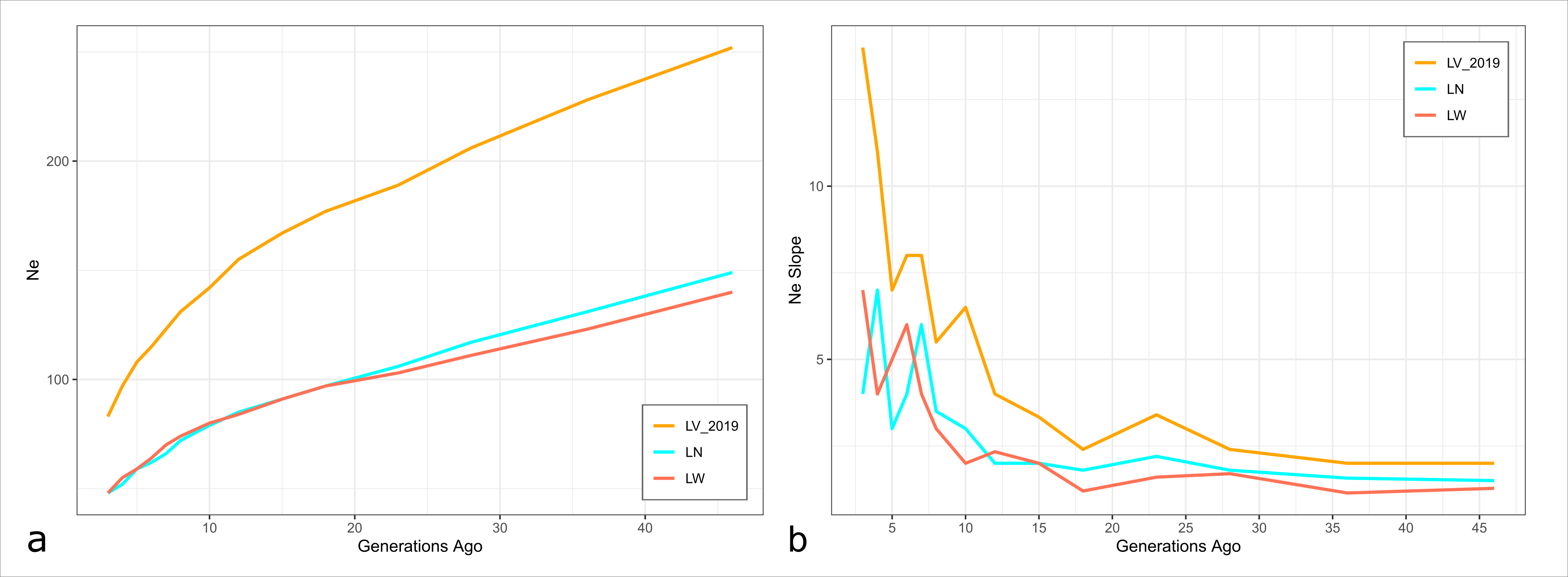

Supplement: Supplementary file 1 [file animals-14-01629-s001.zip › Fig2_Ne_Neslope.png]

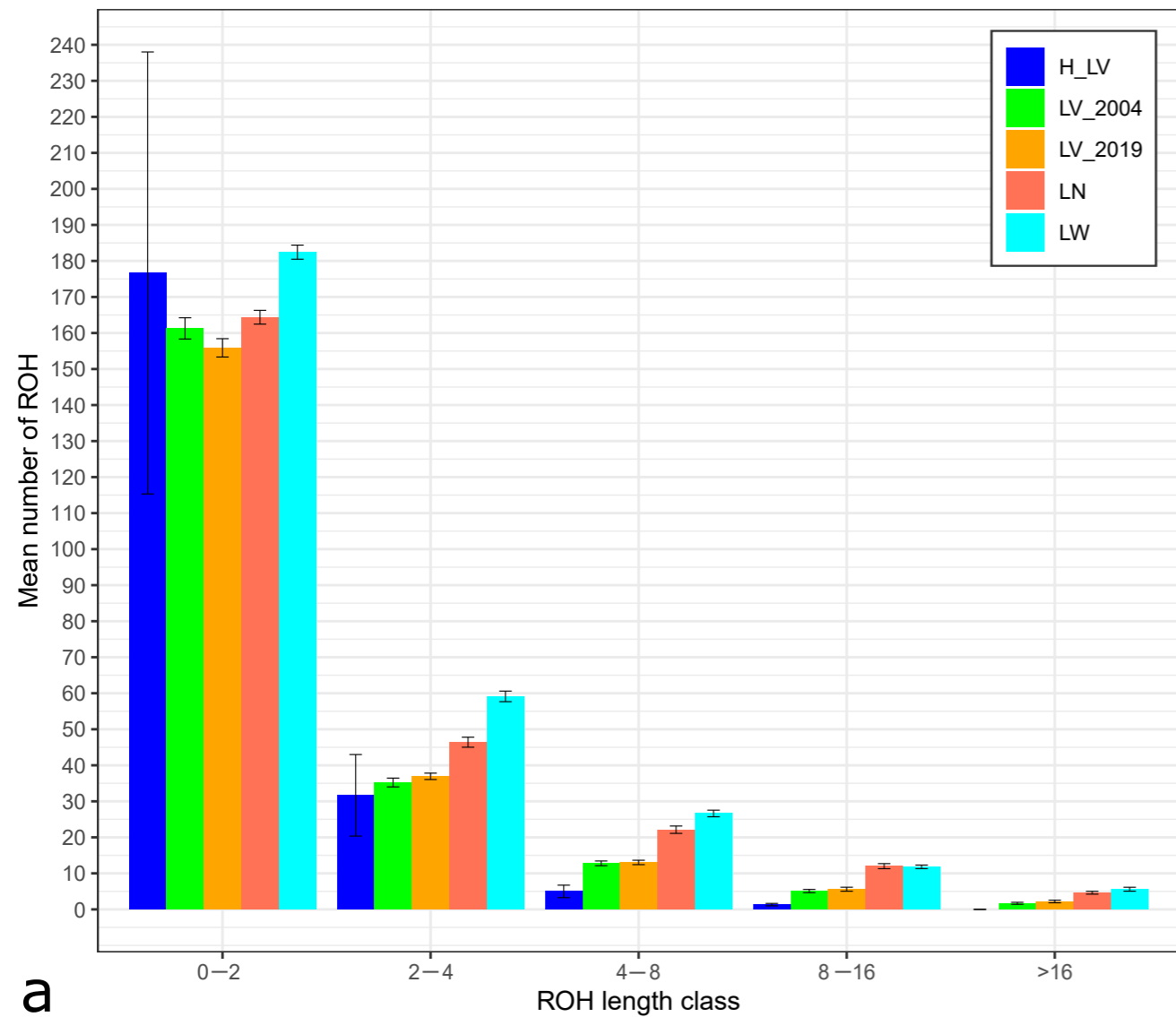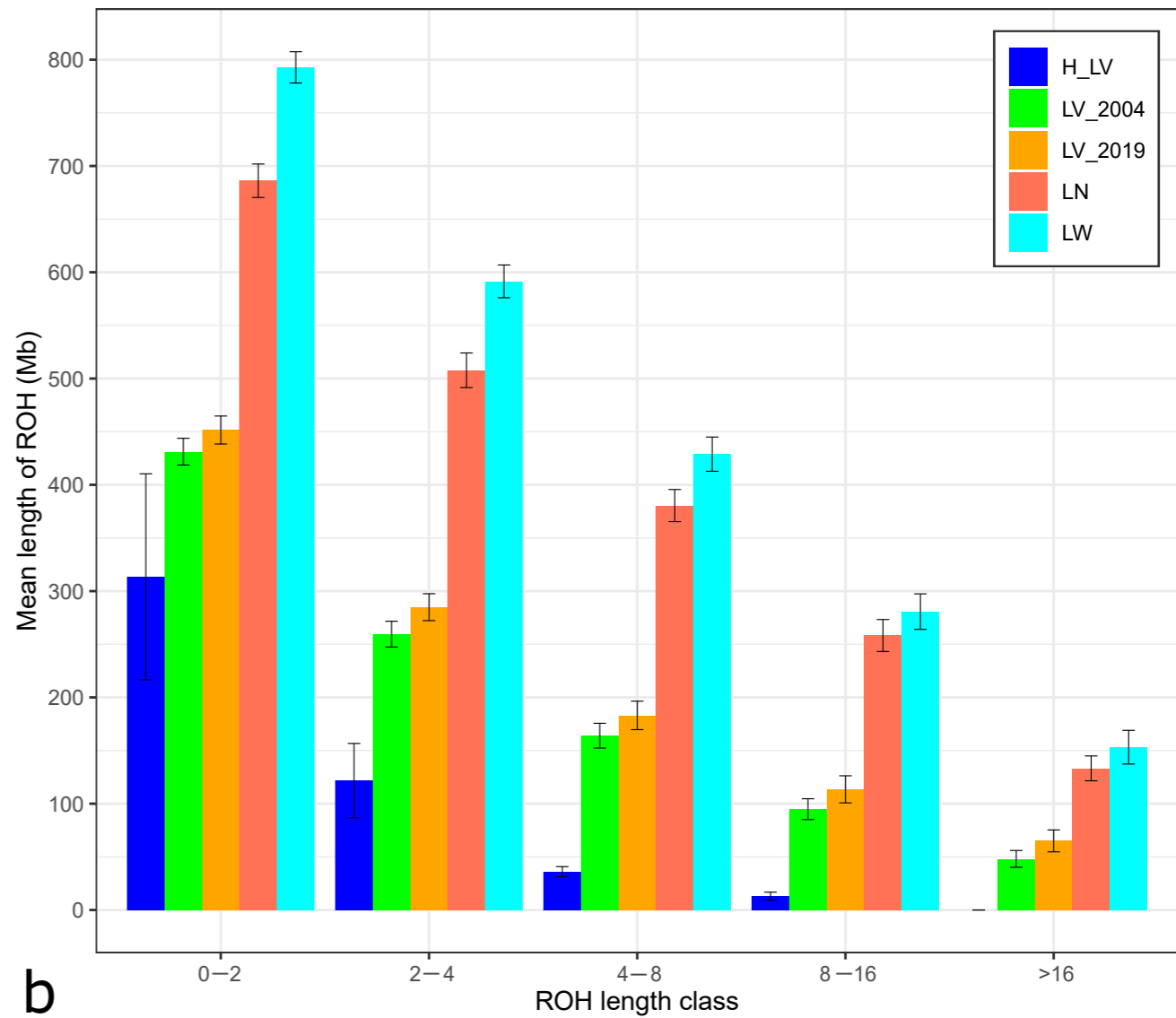

Supplement: Supplementary file 1 [file animals-14-01629-s001.zip › Fig3_ROH_number_length.pdf]

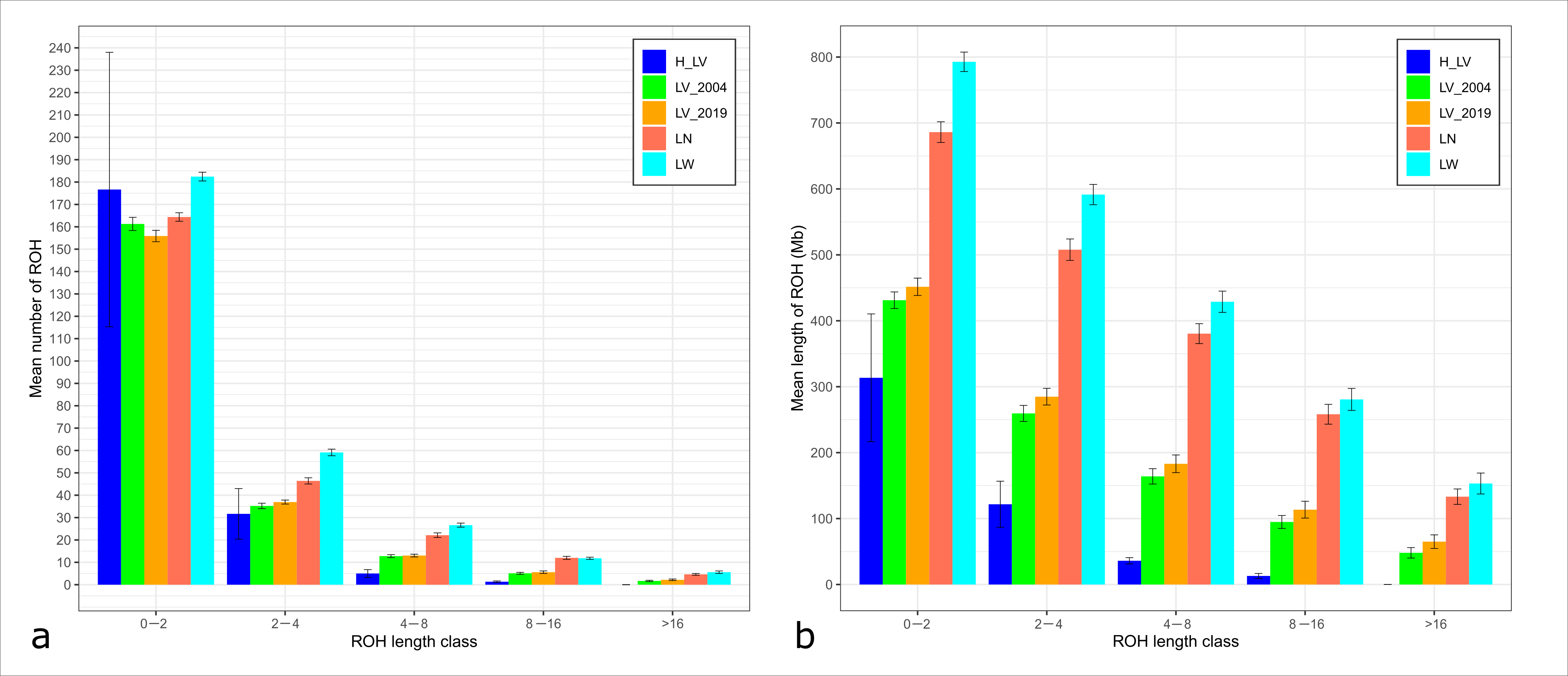

Supplement: Supplementary file 1 [file animals-14-01629-s001.zip › Fig3_ROH_number_length.png]

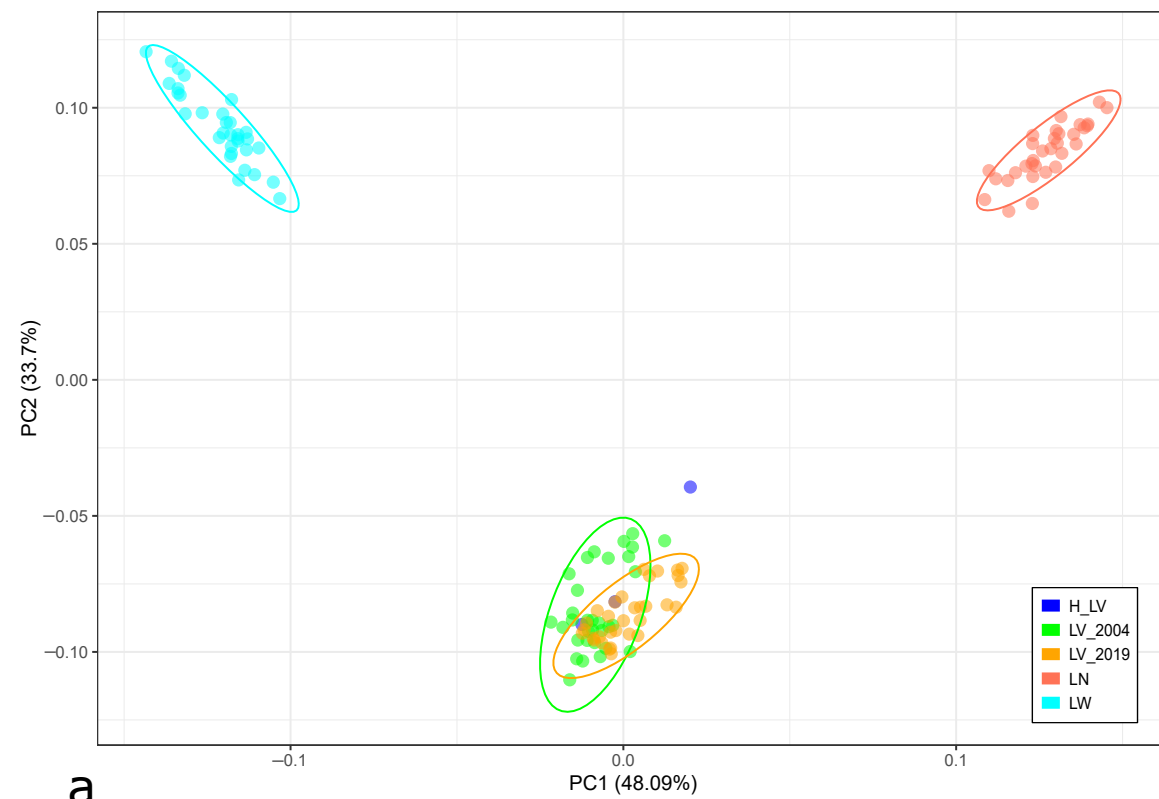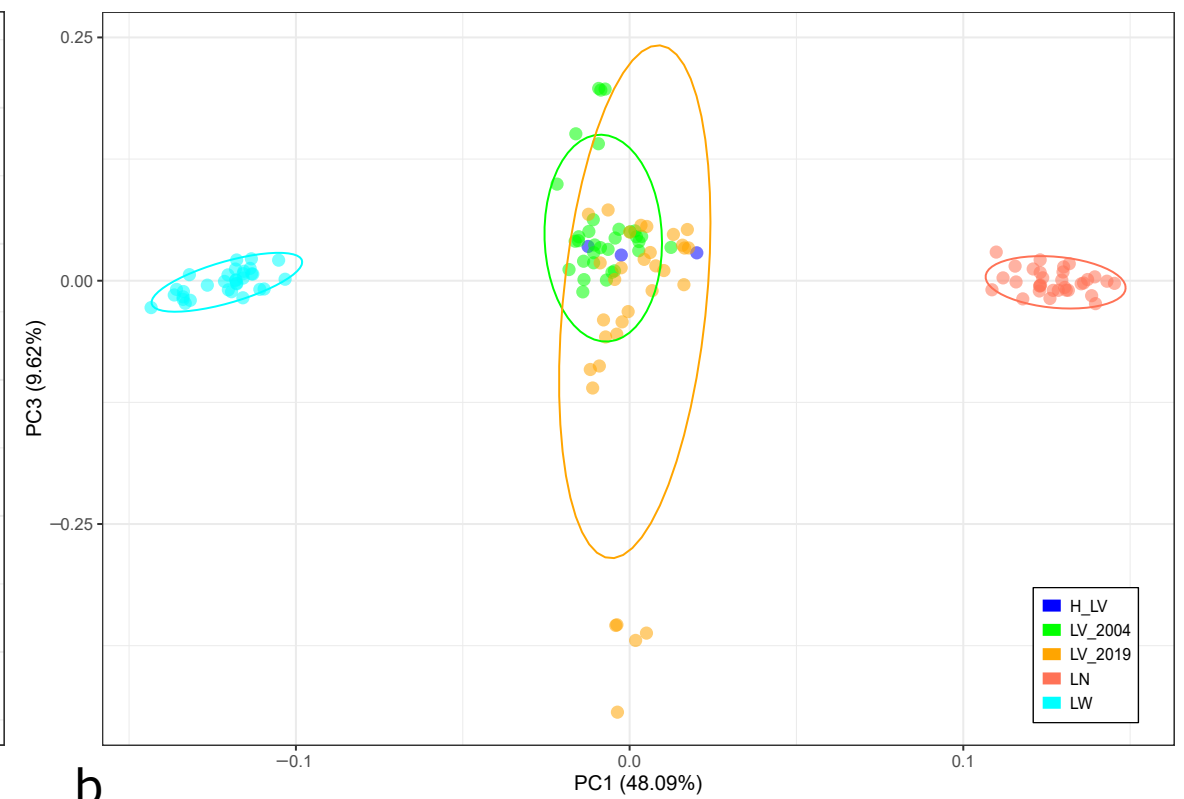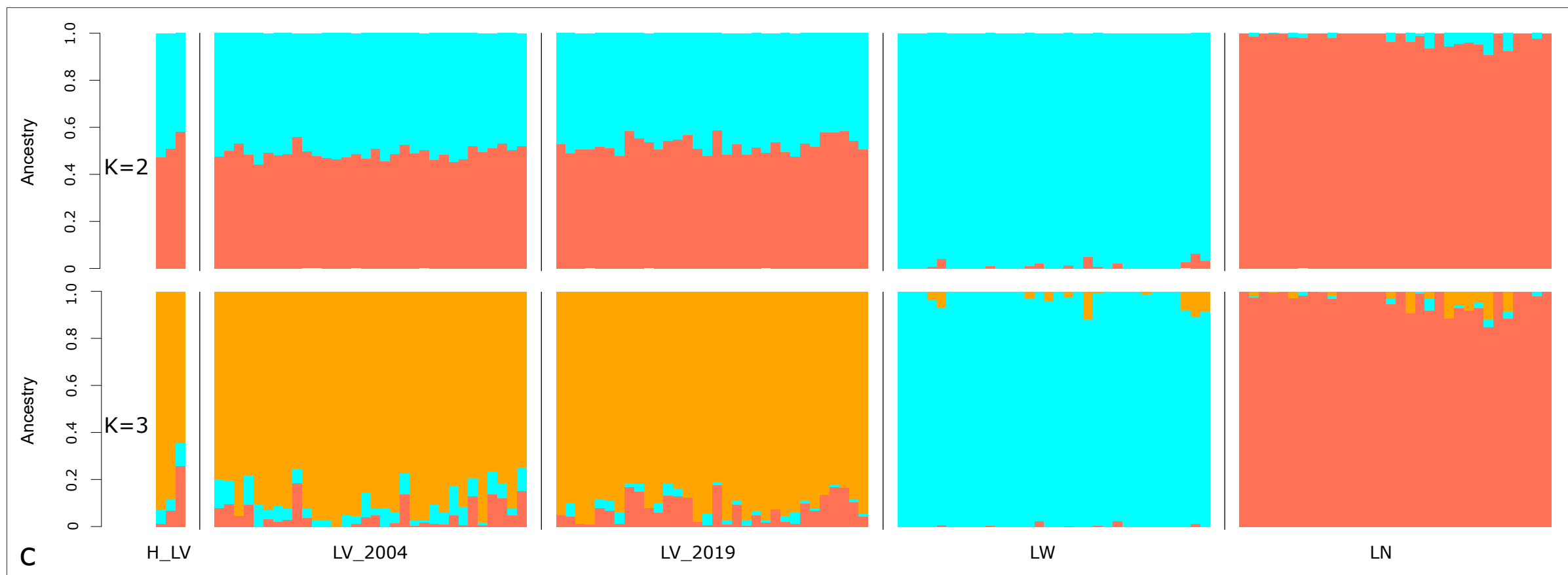

Supplement: Supplementary file 1 [file animals-14-01629-s001.zip › Fig4_PCA_Admixture.pdf]

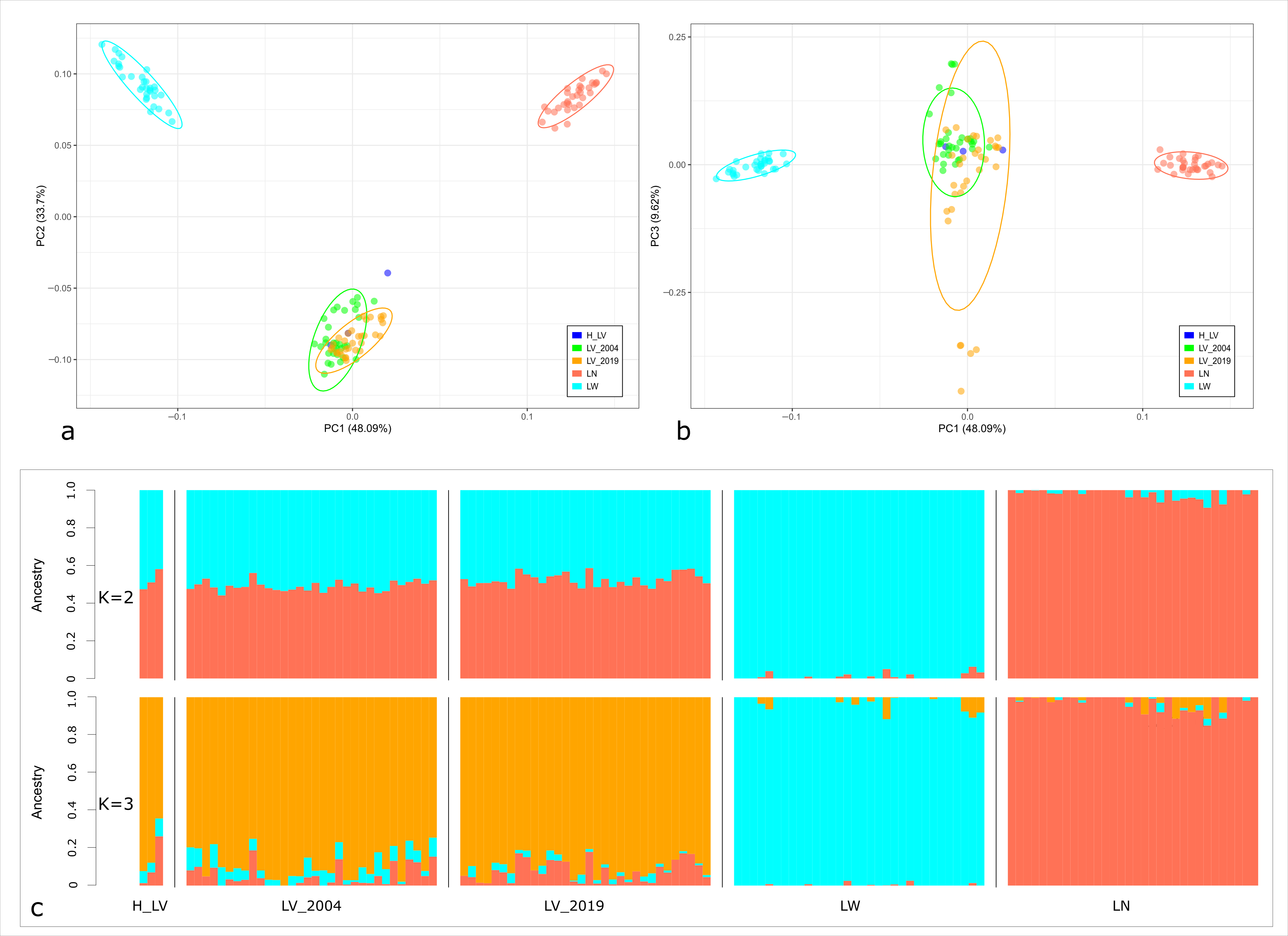

Supplement: Supplementary file 1 [file animals-14-01629-s001.zip › Fig4_PCA_Admixture.png]

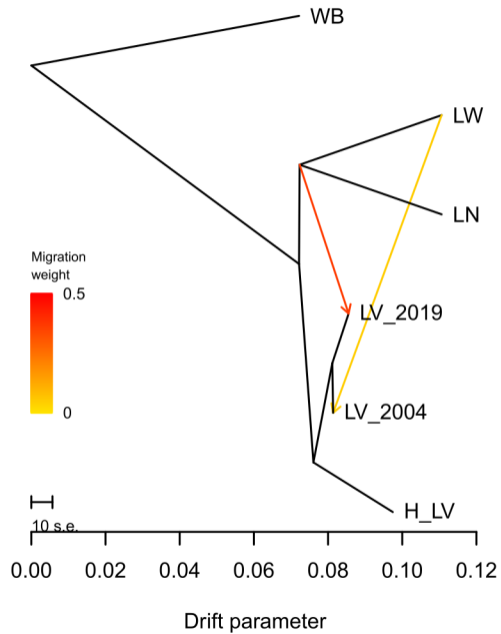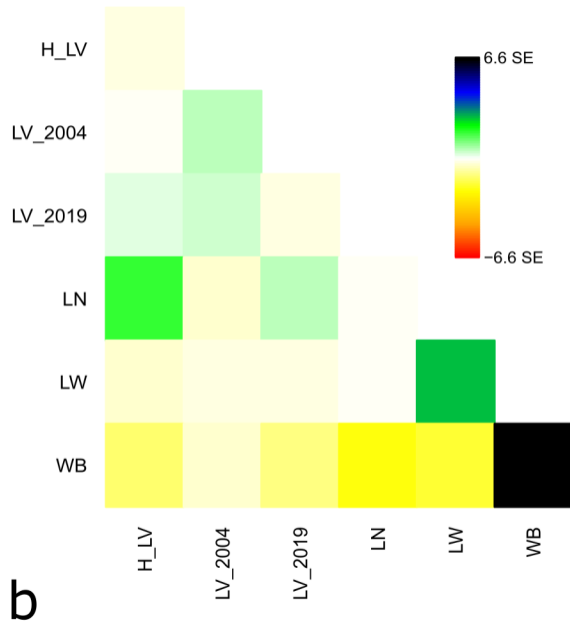

Supplement: Supplementary file 1 [file animals-14-01629-s001.zip › Fig5_tree_res_2m.pdf]

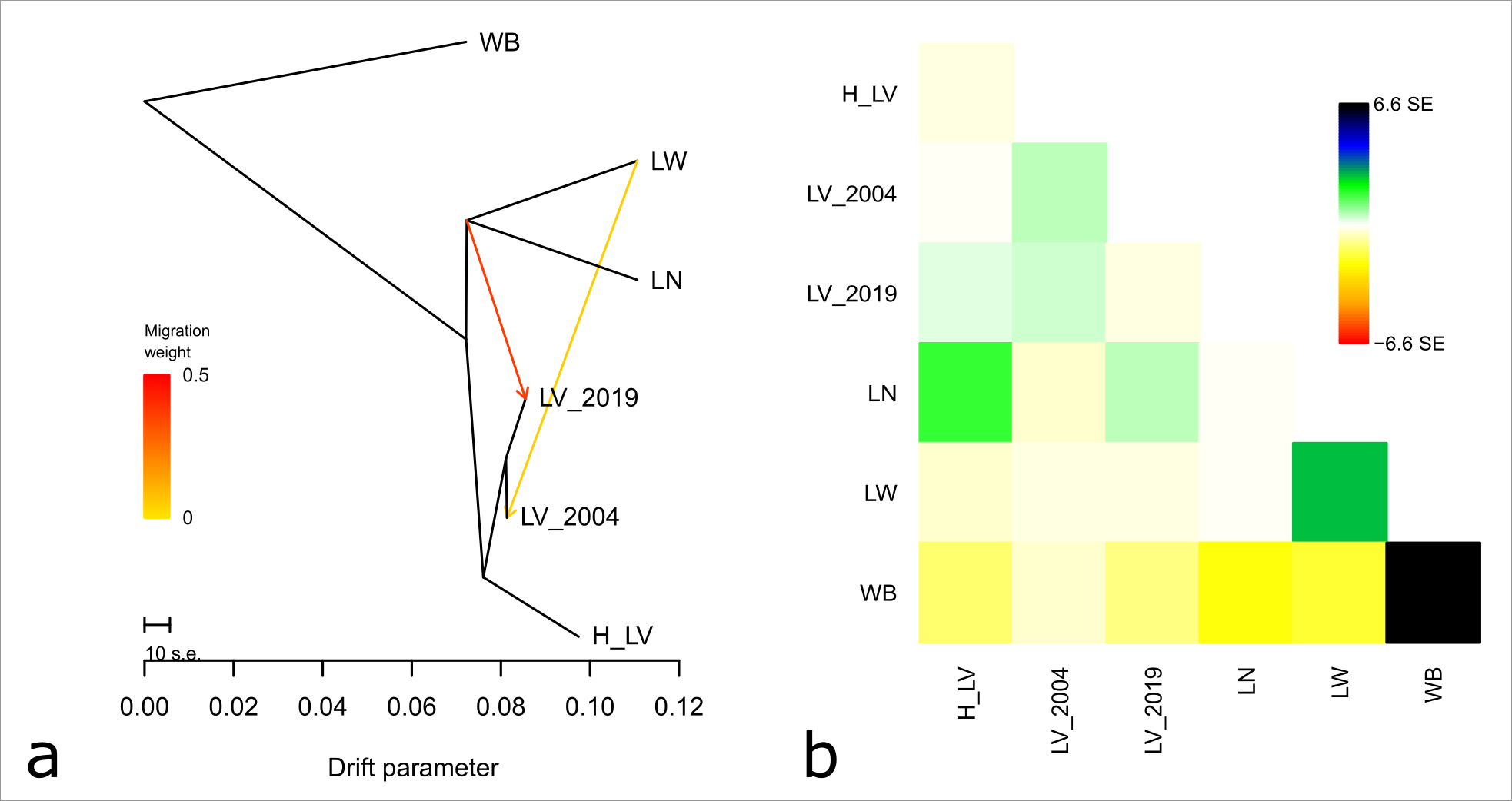

Supplement: Supplementary file 1 [file animals-14-01629-s001.zip › Fig5_tree_res_2m.png]
